# Ginsenoside Rg1 alleviates repeated alcohol exposure-induced psychomotor and cognitive deficits

**DOI:** 10.1186/s13020-020-00325-x

**Published:** 2020-05-07

**Authors:** Lu Huang, Zhuang Peng, Cong Lu, Ying Chen, Jing-wei Lv, Meng Qin, Duan-fang Liao, Xin-min Liu, Zhe Shi

**Affiliations:** 1 Division of Stem Cell Regulation and Application, Key Laboratory for Quality Evaluation of Bulk Herbs of Hunan Province, Hunan University of Chinese Medicine, Changsha, 410208 Hunan China; 2 Guangdong-Hongkong-Macau Institute of CNS Regeneration, Ministry of Education CNS Regeneration Collaborative Joint Laboratory, Jinan University, Guangzhou, 510632 China; 3 Research Center for Pharmacology and Toxicology, Institute of Medicinal Plant Development (IMPLAD), Chinese Academy of Medical Sciences and Peking Union Medical College, Beijing, 100193 China; 4 Institute of Chinese Materia Medica, China Academy of Chinese Medical Sciences, Beijing, 100700 China; 5 College of Life Science and Technology, Beijing University of Chemical Technology, Beijing, 100029 China

**Keywords:** Ginsenoside Rg1, Repeated alcohol exposure, Psychomotor and cognitive deficits, Excitatory glutamatergic transmission, NR2B containing NMDARs

## Abstract

**Background:**

Chronic alcohol consumption disrupts psychomotor and cognitive functions, most of which are subserved by the dysfunction of hippocampus. Dysregulated excitatory glutamatergic transmission is implicated in repeated alcohol induced psychomotor and cognitive impairment. Ginsenoside Rg1, one of the main active ingredient of the traditional tonic medicine Panax ginseng C.A. Meyer (Araliaceae), has been used to treat cognitive deficits. Particularly, Rg1 has been demonstrated to improve hippocampus-dependent learning in mice and attenuate glutamate-induced excitotoxicity in vitro. Thus, in the present research, we sought to investigate the therapeutic effects of Ginsenoside Rg1 on repeated alcohol induced psychomotor and cognitive deficits in hippocampal-dependent behavioral tasks and unravel the underpinnings of its neuroprotection.

**Methods:**

Male ICR (CD-1) mice were consecutively intragastrically treated with 20% (w/v) alcohol for 21 days. Then, behavior tests were conducted to evaluate repeated alcohol induced psychomotor and cognitive deficits. Histopathological changes, and biochemical and molecular alterations were assessed to determine the potential neuroprotective mechanism of Rg1.

**Results:**

The results suggested that Rg1, at the optimal dose of 6 mg/kg, has the potential to ameliorate repeated alcohol induced cognitive deficits by regulating activities of NR2B containing NMDARs and excitotoxic signaling.

**Conclusion:**

Our findings further provided a new strategy to treat chronic alcohol exposure induced adverse consequences.

## Background

Alcohol-use disorders are among the most disabling disease categories for the global burden of disease [1, 2]. In humans, chronic alcohol consumption results in alcoholism and leads to brain shrinkage and loss of nerve cells at specific brain regions via an excitotoxic and oxidative mechanism, which has been regarded as the main pathogenic factor for neurodegeneration [3–5]. Many individuals diagnosed with alcoholism have been reported to exert measurable chronic cognitive impairment [6–8]. Excessive alcohol consumption disrupts cognitive functions in a battery of behavioral tasks in both clinical and experimental studies [9–11], most of which are subserved by the dysfunction of hippocampus [12, 13]. The hippocampus is critical in encoding diverse features of experiences such as spatial locations, landmarks, visual features of the environment, goal locations, conditioned stimuli, and sequences of events [14]. Hippocampal-dependent cognitions are particularly susceptible to the deleterious effects of chronic alcohol exposure, which can result in aberrant structural and functional changes [15–18].

Chronic alcohol consumption leads to elevated levels of extracellular glutamate and triggers excessive activation of various glutamatergic receptors [19–21]. *N*-methyl-d-aspartate receptors (NMDARs) are not only pivotal regulators in normal physiological processes in the central nervous system (CNS), but also important target of alcohol [22]. Chronic exposure to alcohol induces expression and functional alterations of NMDARs [23, 24]. Therefore, regulating the activity of NMDAR signaling could be an effective way to rescue chronic alcohol exposure induced neuronal dysfunction.

Ginsenoside Rg1, one of the main active ingredient of the traditional tonic medicine *Panax ginseng* C.A. Meyer (Araliaceae), has been used to treat cognitive deficits with neuroprotection, anti-oxidative stress, anti-apoptosis, anti-inflammation and neurotrophic properties [25–29]. Particularly, Rg1 has been demonstrated to improve hippocampus-dependent learning in mice and attenuate glutamate-induced excitotoxicity in vitro [30–32]. Thus, we hypothesized that Ginsenoside Rg1could exert beneficial effects on chronic alcohol exposure induced cognitive deficits.

In the present research, we sought to investigate the therapeutic effects of Rg1 on repeated alcohol exposure (RAE) induced psychomotor and cognitive deficits in hippocampal-dependent behavioral tasks and unravel the underpinnings of its neuroprotection.

## Methods

### Animals

Eight to ten weeks old male ICR (CD-1) mice were obtained from Vital River (Beijing, China). They were group-housed under controlled environmental conditions (25 °C and 50–70% humidity) with food and water ad libitum. All mice were acclimatized to a 12-h light/dark cycle (lights on at 7:00 a.m. and lights off at 7:00 p.m.). The animal experimental procedures were approved by the Animal Ethics Committee of Institute of medicinal plant development (IMPLAD), CAMS & PUMC and were conducted strictly according to the National Institutes of Health Guide for the Care and Use of Laboratory Animals (NIH Publications No. 8023, revised 1978).

### Drugs and treatment schedule

Mice were assigned to five groups (Control, Alcohol, Rg1-3 mg, Rg1-6 mg and Rg1-12 mg, n = 12 each) in a quasi-random manner after a 3 days acclimatization. Ginsenoside Rg1, purchased from Chengdu Herbpurify (Sichuan, China), was daily intragastrically administrated at the dose of 3 mg/kg, 6 mg/kg and 12 mg/kg with an intragastric tube in the Rg1 treatmnt groups for 14 days prior to corresponding alcohol treatment and throughout the experiment. Mice in the control and alcohol group respectively received isovolumetric normal saline with an intragastric tube as well. From day 15, all mice except in the control group were daily intragastrically administrated alcohol (20% w/v in isotonic saline) at a dose of 3.4 g/kg until the end of behavioral tests to mimic repeated alcohol exposure.

### Behavioral procedures

#### Locomotor activity

Thirty minutes after alcohol treatment, each mouse was initially situated at the center of the tank to freely explore the environment for 3 min. An overhead video camera was used to record the movements, and the total distances traveled in the following 10 min was analyzed by image analyzer software.

#### Object location recognition (OLR) test

The OLR test was used to evaluate teh recognition memory, which has been described in detail in our previous research [33]. In brief, during a 3-days habituation period, mice were allowed to explore the environment freely in the arena with no objects presented for 10 min each day. On the fourth day, mice were initially placed in the arena where presented two copies of novel objects (A1 and A2) and allowed to explore (5 min per trial) freely during the familiarization period. After a 30-min interval, mice returned to the arena for the test trial, during which one of the original objects were moved into new location (‘novel’) and the other remained in the previous position (‘familiar’). Objects and their placement were presented in a counter-bias manner to avoid positional preferences. A video camera was used to record the exploratory behavior. The behavioral changes were analysed and scored by two double-blind sophisticated experimenters. The discrimination index (DI) formula used for scoring the recognition memory of each animal is as follows: DI = (TN − TF)/(TN + TF). TN, exploration time on the object changed location; TF, exploration time on the object unchanged location.

#### Morris water maze (MWM) test

The MWM test was administered subsequently to evaluate spatial learning and memory. As previously reported in detail [33], the equipment consists of a stainless steel tub which was divided into four equal quadrants. Make sure the water temperature was maintained at 25 °C. A hyaline platform (6 cm in diameter and 15 cm in height) was submerged 1 cm below the surface and placed in one of the quadrant (e.g. SE). An overhead video camera was used to record the swimming activity and the data was analyzed by image analyzer software. Thirty minutes after drug treatment, mice were subjected to find the submerged platform three trials per day for four consecutive days, with each trial having a ceiling time of 90 s. The escape latency was recorded according to the time for mice to find the submerged platform. In the probing test day, the platform was removed. Each mouse was freed from quadrant (e.g. NW) opposite to which the platform used in (the target quadrant). Every animal was tested only once and the time spent in the target quadrant was recorded until 90 s.

#### Tissue preparation

After finishing behavioral tests, mice were anesthetized and transcardially perfused with ice-cold saline immediately and half number of mice in each group were followed by 4% paraformaldehyde to fix for histological analysis, the rest of the mice brains were quickly removed and placed on ice in order to dissect the hippocampus prepares for total protein extraction.

#### Histopathology analysis

Before being dehydrated, the brains were removed and post-fixed overnight in paraformaldehyde, then embedded in paraffin. Brains were cut into 10 μm thick sections in the coronal plane and stained with hematoxylin and eosin. The hippocampus CA1, CA3, and DG subregions in brain sections were used to observe pathological alterations.

#### Western blotting

After extracting total proteins, the protein concentration was assayed by a BCA protein assay kit. The protein extracts were subjected to 8% or 12% SDS-PAGE (CWBIO, China) and transferred onto a polyvinylidene difluoride (PVDF) membranes. The membrane was treated for 1 h with blocking solution (5% skim milk in TBST) and incubated at 4 °C overnight with the primary rabbit monoclonal antibodies respectively (NR1, CST, 1:1000; NR2B, CST, 1:1000; m-calpain, Abcam, 1:500; STEP Antibody (23E5), Novus Biologicals, 1:1000; p-p38, Abcam, 1:500 and β-actin, CST, 1:2000). The next day, after incubation with secondary goat-anti-rabbit antibody (1:1000, CST) for 1 h, the enhanced chemiluminescence (ECL) was then utilized to visualize immunoreactive proteins and the signals were quantified by densitometry with a Western blotting detection system (Quantity One, Bio-Rad, USA).

#### Biochemical analysis

The concentration of glutamate (Glu) in the hippocampus was determined by an LC–MS/MS method previously described in detail [14]. After weighed the tissues, ice-cold aqueous acid was prepared to homogenize the tissues, then precipitate protein in formic acid. The supernatant was collected after centrifugation and mixed with an internal standard solution (300 μg/mL DHBA), then collected 50 μl of the mixture to analyze by LC–MS/MS system. Agilent 1200 HPLC system (Palo Alto, CA, USA), an Applied Biosystem 3200 Q-Trap mass spectrometer (Foster City, CA, USA) and an electrospray ionization source constitute LC–MS/MS instrument. The mobile phase consisted of 6 mM ammonium formate in acetonitrile–water (67.5:32.5, pH 5.50). The detection limit and quantification ranged from 0.96 to 24.48 nmol/L and 3.42 to 244.82 nmol/L. The quantification of Glu was according to the ratios of the peak areas of the analyte versus the internal standard.

### Statistical analysis

All analyses were performed by SPSS version 23.0 for Mac (IBM, USA). All data were represented as the mean ± SEM. Data were analyzed with a one-way analysis of variance (one-way ANOVA) or repeated-measures analysis of variance (RM ANOVA) where statistically appropriate. Post hoc multiple pairwise comparisons were made with the LSD comparisons test after ANOVA when significant effects were detected [34]. The analysis results were only presented when a significant difference was observed. Statistical significance was defined as *P* < 0.05 [35].

## Results

### Rg1 alleviated RAE induced cognitive deficits in hippocampal-dependent behavioral tasks

**Fig. 1:**
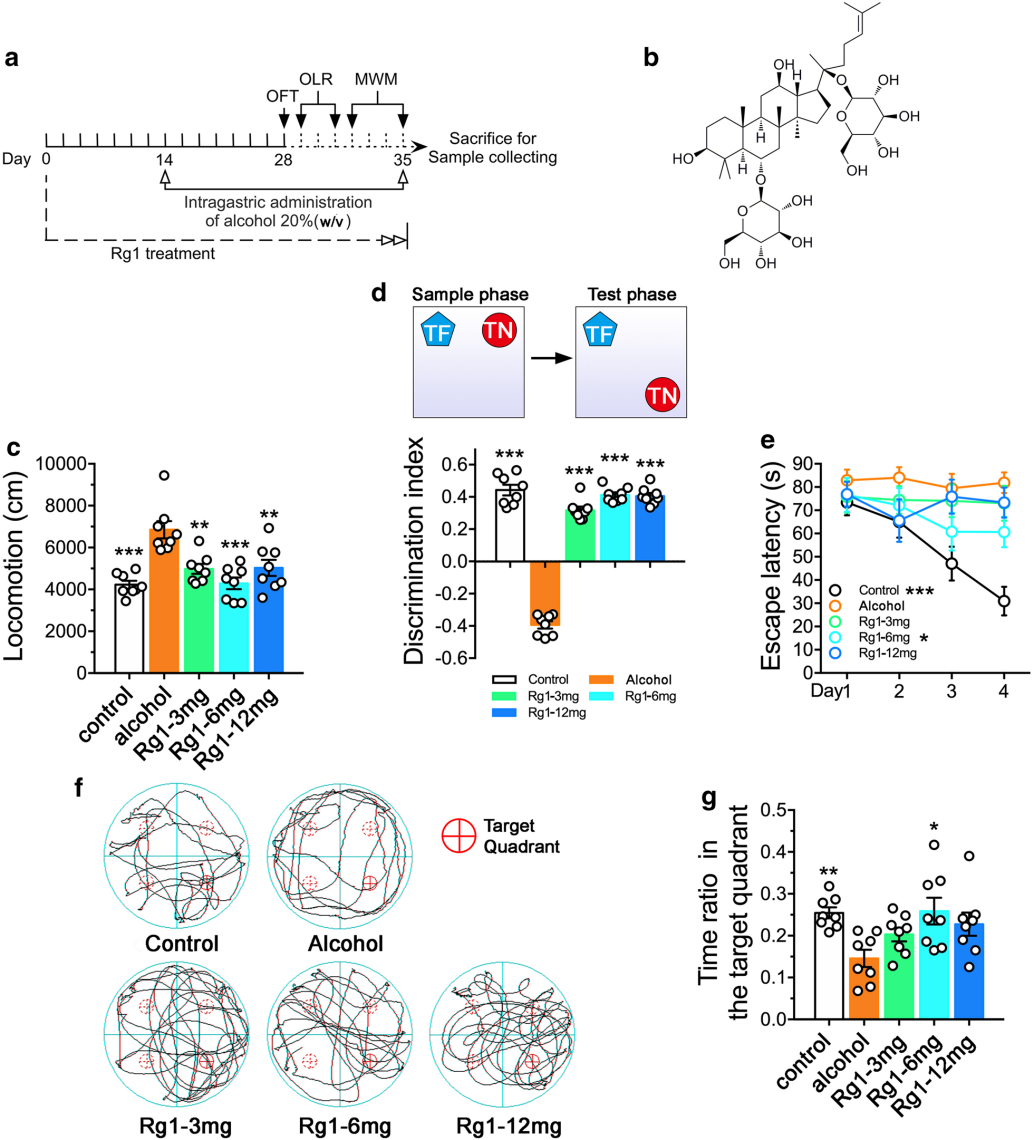
Ginsenoside Rg1 treatment alleviated repeated alcohol-induced cognitive deficits. **a** A schematic illustration of the experimental manipulation. **b** chemical structure of ginsenoside Rg1. **c** The open field test showed that Rg1 pretreatment decreased RAE-induced psychomotor response. **d** The object location recognition test demonstrated that the impaired recognition ability was recovered after Rg1 treatment. **e**–**g** Mice received Rg1 pretreatment showed better spatial navigation and orientation abilities in the reference learning and memory retention tasks. (*P < 0.05, **P < 0.01, ***P < 0.001, compared with alcohol group; n = 8 per group)

To determine RAE induced cognitive deficits, mice were subjected to open field test, object location recognition, and morris watermaze test. Open field test was conducted to assess psychomotor responses of mice after alcohol exposure. As shown in Fig. 1b, hyperactivity was observed in mice received repeated alcohol treatment (F_4,35_ = 11.62, *P *< 0.001). Mice with Rg1-3 mg/kg (*P *< 0.01), 6 mg/kg (*P *< 0.001) and 12 mg/kg (*P *< 0.01) treatment exerted less psychomotor response to alcohol exposure. Moreover, the object location recognition test was conducted to assess the ability to distinguish both object location and features. We noticed that mice in the alcohol group spent more time to explore the location unchanged object (F_4,35_ = 233.4, *P *< 0.001), while mice in the Rg1-3 mg/kg (*P *< 0.001), 6 mg/kg (*P *< 0.001) and 12 mg/kg (*P *< 0.001) treatment groups spent more time to explore the location changed object (see Fig. 1c). Furthermore, the water maze tests were performed to assess the hippocampus-dependent spatial reference memory. As shown in Fig. 1d–f Mice in the alcohol group were obviously retarded to find the invisible platform during the 4 days reference learning task (F_4,115_ = 7.947, *P *< 0.001) and performed worse to retrieve the spatial memory (F_4,35_ = 4.019, *P *< 0.01). Notably, mice pretreated with Rg1 at 6 mg/kg dosage, exerted better spatial navigation (*P *= 0.025) and orientation (*P *= 0.0115) abilities in the reference learning and memory retention tasks.

### Rg1 treatment suppressed RAE-induced neuro-excitotoxicity in the hippocampus

**Fig. 2:**
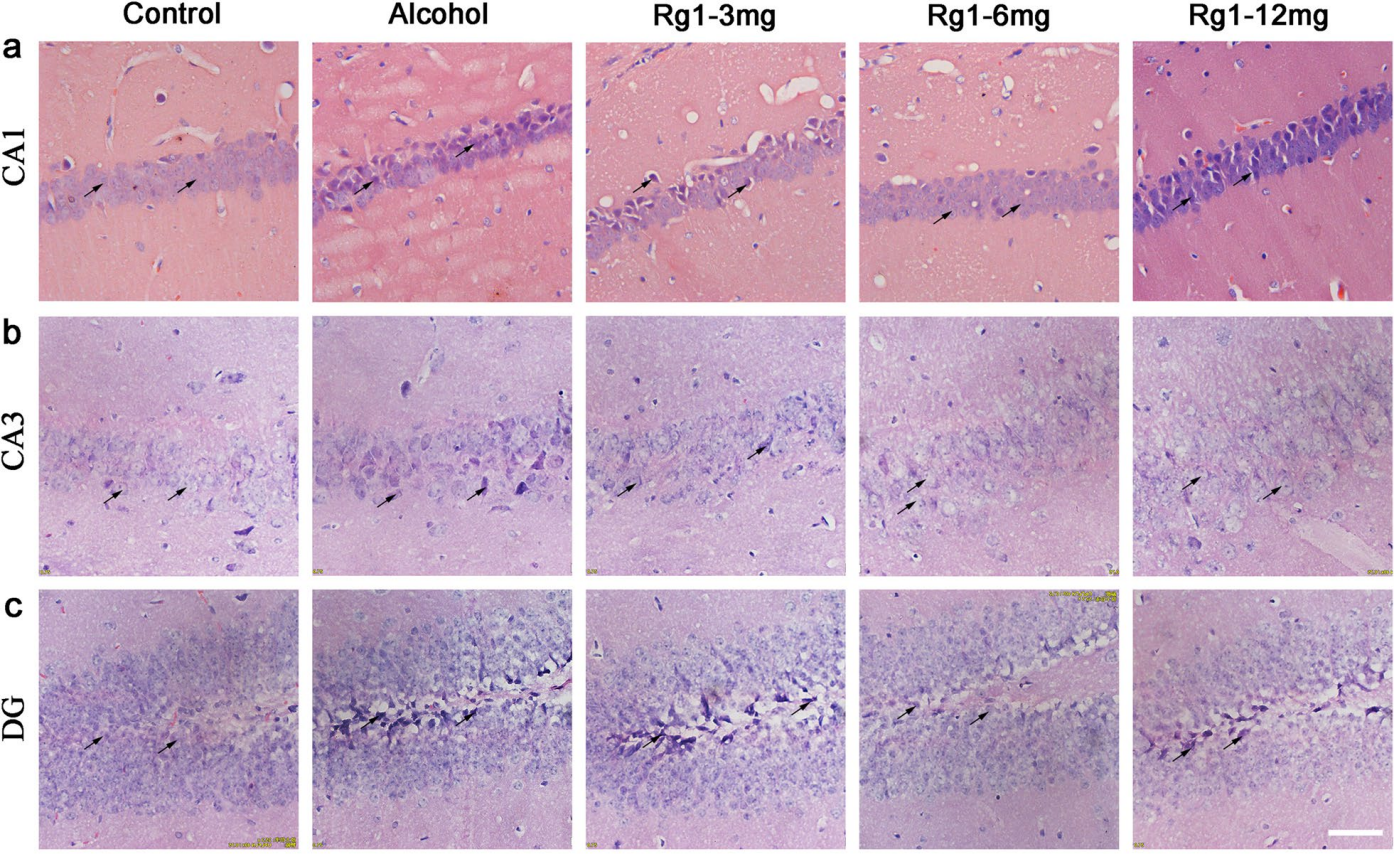
Ginsenoside Rg1 treatment prevented chronic alcohol-induced pathological alteration of neurons in the hippocampus. HE staining of hippocampal neurons in the CA1 (**a**), CA3 (**b**) and DG (**c**) subregions are shown (n = 5 per group, scale bar = 50 μm)

Then, we determined whether RAE-induced psychomotor and cognitive deficits were associated with pathological alterations in the hippocampus. We conducted H&E staining in hippocampal slices. The HE staining could visually show histological changes in neurons. As shown in Fig. 2a–c, in the control mice, neurons in the CA1, CA3 and DG subregions of the hippocampus were round or oval in shape and the nuclei were clear. After RAE, numerous impaired neurons with karyopyknosis, cell gaps, and debris were observed in the CA1, CA3 and DG subregions of the hippocampus in the alcohol group. However, Rg1 treatment significantly reversed RAE-induced morphological alterations in varying degrees. Notably, Rg1 treatment at the dose of 6 mg/kg showed a better curative effect to protect neurons from alcohol insult.

### Rg1 treatment reduced glutamate spillover and NR2B-containing NMDARs activation in the hippocampus

**Fig. 3:**
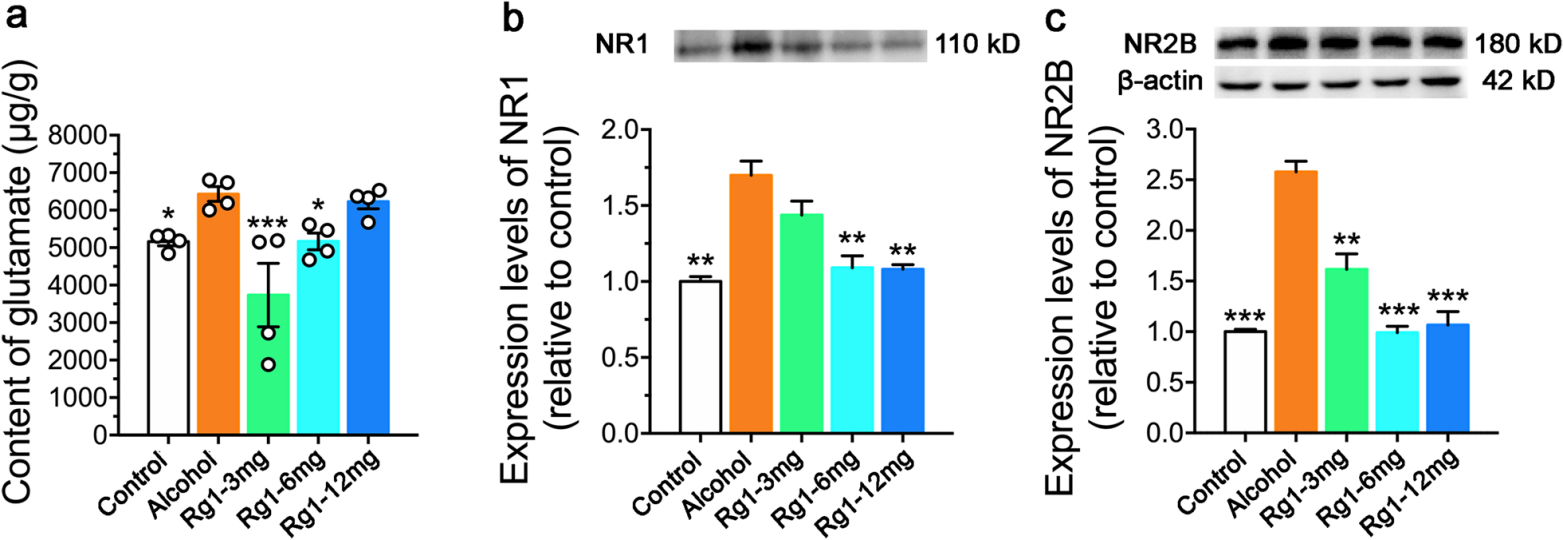
Ginsenoside Rg1 treatment reduced glutamate spillover and NR2B-containing NMDARs activation in the hippocampus. **a** Alterations in glutamate content. Expression level changes in NR1 (**b**) and NR2B (**c**) in the hippocampus (*P < 0.05, **P < 0.01, ***P < 0.001, compared with alcohol group; n = 4 per group in glutamate content examination and n = 3 per group in western blot assay)

To determine whether the observed pathological changes in the hippocampus were related to the dysregulation of excitatory glutamatergic transmission. We detected the changes of glutamate content in the hippocampus by a LC–MS/MS method. As shown in Fig. 3a, glutamate content was obviously higher after RAE (F_4,15_ = 6.756, *P *= 0.037). Rg1 treatment at the doses of 3 mg/kg (*P *< 0.001) and 6 mg/kg (*P *= 0.047) significantly reduced glutamate levels in the hippocampus. Moreover, RAE induced excessive glutamate release triggers the activation of NMDARs. We noticed that RAE significantly elevated the expression levels of NR1. Since NR1 is obligatory in the heterotetramer, we further determined the positive correlation between the expression levels of NR1 and NR2B. Thus, it is conceivable RAE could excessively activate NR2B containing NMDARs. Notably, Rg1 treatment inhibited NR2B activation at the dose of 6 mg/kg and 12 mg/kg. All things considered, Rg1 treatment at the dose of 6 mg/kg displayed the optimal pharmacological activity in reducing glutamate spillover and inhibiting NR2B-containing NMDARs activation.

### Rg1 exerts neuroprotective effects via suppressing extrasynaptic NMDARs-mediated excitotoxic signaling

**Fig. 4:**
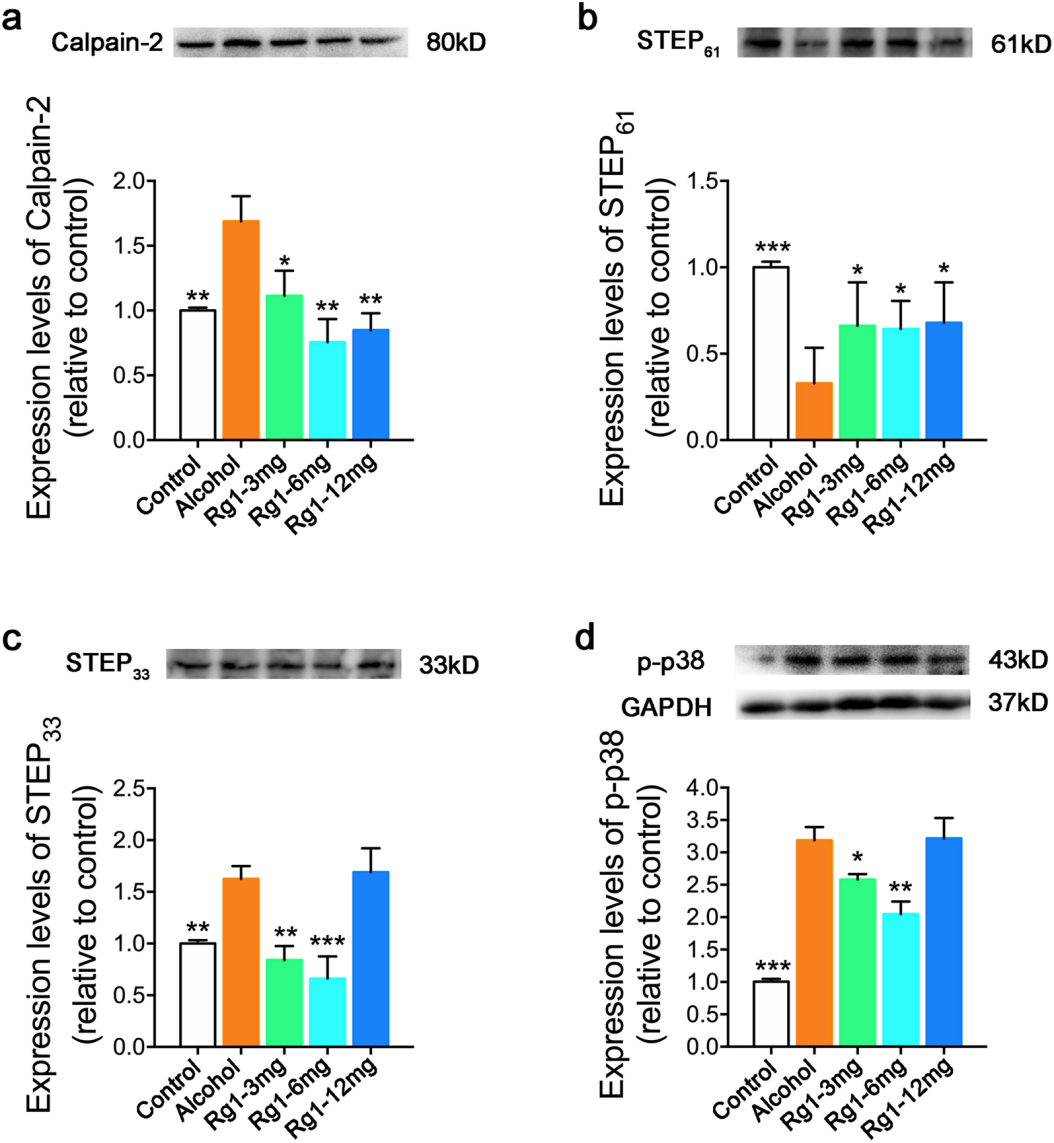
Ginsenoside Rg1 exerts neuroprotective effects via suppressing extrasynaptic NMDARs-mediated excitotoxic signaling. Expression level changes in calpain-2 (**a**), STEP61 (**b**), STEP33 (**c**) and p-p38 MAPK (**d**) in hippocampus are shown (*P < 0.05, **P < 0.01, ***P < 0.001, compared with alcohol group; n = 3 per group)

To reveal the potential mechanism by which Rg1 exerts its neuroprotection, extrasynaptic NMDARs mediated excitotoxic signaling was assessed. As shown in Fig. 4a–d, calpain-2 expression was obviously elevated after RAE. Subsequently, STEP_61_ converted to STEP_33_ in repeated alcohol-treated mice. Increased STEP_33_ expression ultimately activated the phosphorylation of p38 MAPK. Intriguing, Rg1 treatment significantly reversed extrasynaptic NMDARs mediated excitotoxic signaling in a dose-dependent manner. Rg1 treatment suppressed the activation of calpain-2 and the transformation of STEP_61_ to STEP_33_, which effectively inhibited the phosphorylation of p38 MAPK. Thus, it can be inferred that Rg1 protected hippocampal neurons by suppressing calpain-2/STEP/p38 excitotoxic cascade.

## Discussion

The consumption pattern of alcohol is critical to determine the nature and extent of pathological and behavioral consequences. Repeated daily administration of alcohol is reliable to induce compulsive alcohol seeking, psychomotor and cognitive deficits [36–38]. Consistently, in the present study, we found that RAE disrupted psychomotor and cognitive functions in several hippocampus-dependent behavioral tasks. Excessive alcohol consumption leads to impairments in behavioral control, learning, memory and executive functions attributed, at least partially, to the integrity of the hippocampus [39–41]. Hippocampus is essential to transmitting circumstantial and contextual information via glutamatergic afferents to the prefrontal cortex and ventral striatum, and retrieves previous experience to guide behavior [14, 42].

Chronic alcohol exposure was reported to tolerate to the sedative effects of alcohol and result in withdrawal hyperexcitability [43, 44]. Consistently, as shown in Fig. 1b, locomotor activities of mice were significantly increased after RAE. Rg1 treatment significantly alleviated psychomotor responses to alcohol, which is critical for the development of addiction [45]. Moreover, water maze tests were used to assess spatial reference learning and memory [46]. Hippocampus has been well demonstrated to play a pivotal role in spatial memory [47, 48]. In the reference learning task, animals are required to find an invisible platform placed in one of four quadrants. They had to learn the location using extrinsic cues and retain this information in the memory retention test. Mice received repeated alcohol administration were obviously unable to find the invisible platform during the 4 days reference learning task and performed worse to retrieve the spatial memory (see Fig. 1d–f). Mice pretreated with Rg1 (6 mg/kg) exerted better spatial navigation and orientation abilities in the reference learning and memory retention tasks. Furthermore, hippocampus also involves in recognition ability [49, 50]. The object location recognition task depends on the creature’s natural instinct to explore novel items or a novel location [51]. Hippocampal neurons, particularly in the CA1 subregion, are critically involved in encoding both object location and identity information and hence play a key role in forming object-in-place association [52, 53]. In the test phase, mice received repeated alcohol treatment spent more time to contact with the location unchanged object, which implied the impairment of recognition ability (see Fig. 1c). Rg1 pretreatment significantly ameliorated RAE induced deficit in recognition ability. These data demonstrated that repeated alcohol administration resulted in cognitive deficits in spatial learning and memory, and recognition ability could be effectively reversed by Rg1 treatment. All ginsenosides including Rg1 share the same dammarane-type triterpenoid structural [54]. Abundant evidence has demonstrated the neuroprotective effects of the 20(S)-protopanaxadiol analogues [55]. Based on our finding, it can be inferred that the 20(S)-protopanaxadiol analogues may possess a similar potential effect on repeated alcohol induced psychomotor and cognitive deficits, though the precise mechanism remains to be further determined.

Chronic alcohol exposure leads to excessive release and accumulation of extracellular glutamate, a key excitatory neurotransmitter involved in vital physiological processes in the CNS [19, 21]. Glutamate triggers over-activation and expression of calcium-permeable NMDARs, which leads to adaptive functional changes of these receptors [56–58]. Consequently, in the present research, repeated alcohol administration enhanced glutamate spillover and resulted in pathological changes of neurons in the CA1, CA3 and DG subregions of the hippocampus, which was possibly associated with the over-expression of NR2B-containing NMDARs. Most NMDARs are composed of two essential NR1 and two modulatory NR2 subunits [59]. The number of NMDAR is in a dynamic equilibrium between synaptic, extrasynaptic, and intracellular compartments [60]. The functional or pathological effects of NMDARs are closely related to their locations. Any shift in balance to enhance extrasynaptic NMDAR signaling may be detrimental to neuronal health. Synaptic NMDARs are thought to mediate neuronal plasticity, while extrasynaptic NMDARs are linked to Ca^2+^ regulation and glutamate excitotoxicity [61–63]. Excessive activation of extrasynaptic NMDARs initiates dysregulation of intracellular Ca^2+^ and enhances neuronal susceptibility to excitotoxic damage [64–66]. Activated NR2B-containing NMDARs subsequently leads to calpain activation [67]. Calpains are calcium-dependent proteases that have been implicated in a wide range of pathological states [68]. Distinct isoforms of calpains differentially activated by synaptic and extrasynaptic NMDAR stimulation, with the former activating calpain-1 (μ-calpain) and the latter activating calpain-2 (m-calpain) [69]. We further determined that repeated alcohol administration induced elevation of NR2B-containing NMDARs expression was associated with calpain-2 activation, which indicated that extrasynaptic NMDARs were excessively activated after RAE. Calpain-2, rather than calpain-1, results in proteolysis of downstream striatal-enriched protein phosphatase (STEP) and subsequent activation of p38 mitogen-activated protein kinase (p38 MAPK) [70].

STEP is highly enriched in the striatum, hippocampus, and cortex [71]. It forms a complex with the NMDAR and regulates the responsiveness of NMDAR to ethanol effects in the hippocampus [72, 73]. STEP cleaved from a membrane-associated STEP_61_ isoform into a lower molecular-weight cytosolic-enriched STEP_33_ isoform during excitotoxic assault [74, 75]. In addition, p38 MAPK, which is expressed in extrasynaptic sites and implicated in NMDARs-mediated excitotoxic damage, is negatively regulated by STEP [70, 76]. Thus, reversing calpain-mediated STEP cleavage is sufficient to inhibit NMDARs-dependent p38 MAPK activation and protect neurons from excitotoxic damage. Intriguingly, we noticed that Rg1 treatment significantly reversed extrasynaptic NMDARs mediated excitotoxic signaling in a dose-dependent manner. Rg1 treatment suppressed the activation of calpain-2 and the transformation of STEP_61_ to STEP_33_, which effectively inhibited the phosphorylation of p38 MAPK. Our findings suggested that repeated alcohol administration induced cognitive deficits could be attributed to NMDAR-mediated excitotoxic assault. Rg1 pretreatment significantly protected neurons in the hippocampus by suppressing excitotoxic NMDAR activity.

## Conclusion

**Fig. 5:**
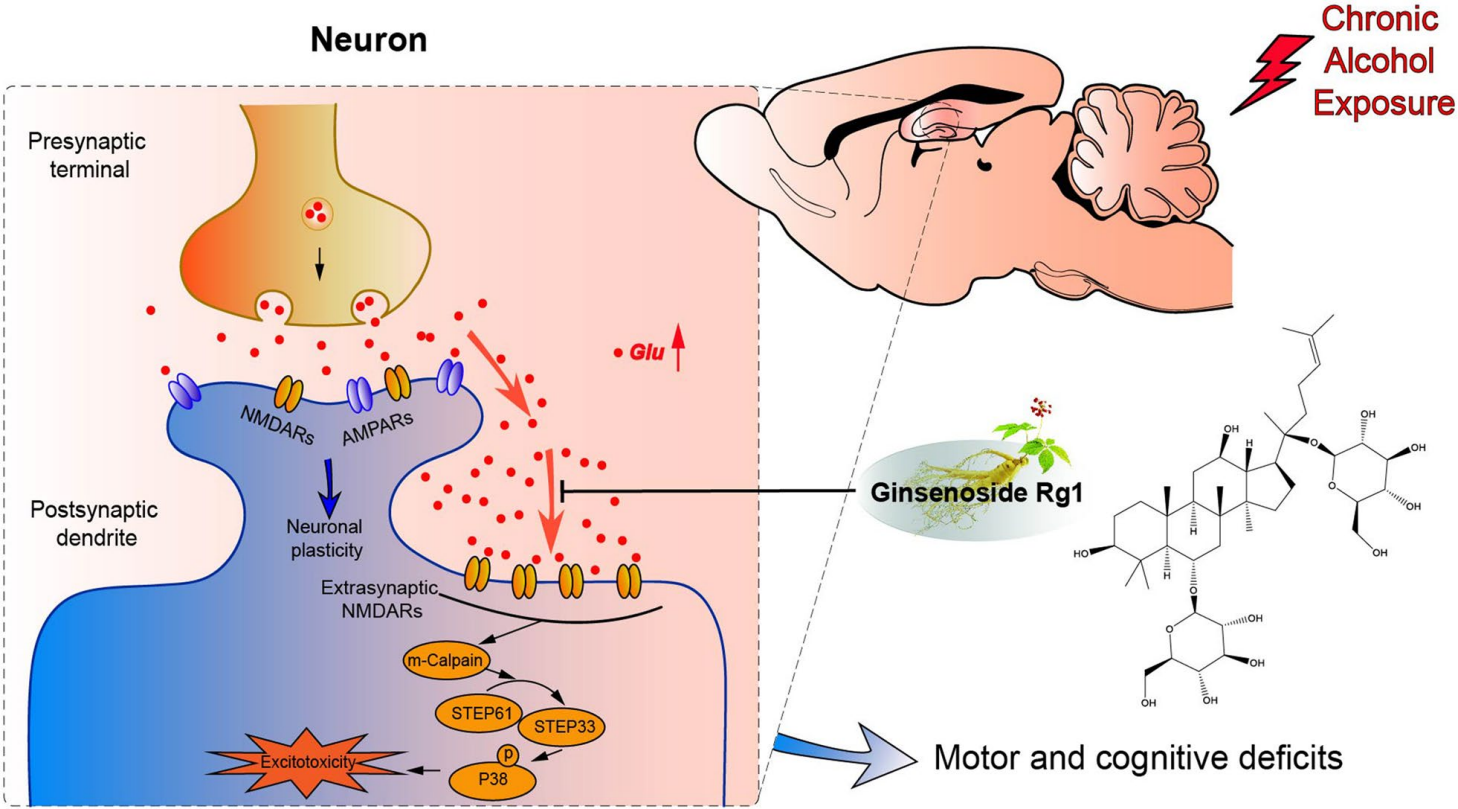
A schematic diagram illustrating the proposed mechanism by which ginsenoside Rg1 alleviated repeated alcohol-induced cognitive deficits. Repeated alcohol exposure results in glutamate spillover and over-activation of extrasynaptic NR2B-containing NMDARs in the hippocampus. Activated extrasynaptic NMDARs initiates calpain-2/STEP/p38 excitotoxic cascade and leads to pathological changes of neurons in the hippocampus. Rg1 pretreatment can effectively ameliorate cognitive deficits via suppressing extrasynaptic NMDARs-mediated excitotoxic assaults

Data from the present research suggested that Rg1, at the optimal dose of 6 mg/kg, has the potential to ameliorate excessive alcohol intake induced cognitive deficits by regulating extrasynaptic NMDARs-mediated excitotoxic signaling (breifly illustrated in Fig. 5). Our findings further provided a new strategy to treat chronic alcohol intoxication induced excitotoxicity and neurodegeneration.

## Data Availability

All the data used to support the findings of this study are available from the corresponding author upon reasonable request.

## Abbreviations

CNS: Central nervous system
Glu: Glutamate
MWM: Morris water maze
NMDARs: *N*-methyl-d-aspartate receptors
OLR: Object location recognition
RAE: Repeated alcohol exposure
p38 MAPK: p38 mitogen-activated protein kinase
STEP: Striatal-enriched protein phosphatase
